# Specific regulation of mechanical nociception by G**β**5 involves GABA-B receptors

**DOI:** 10.1172/jci.insight.134685

**Published:** 2023-07-10

**Authors:** Mritunjay Pandey, Jian-Hua Zhang, Poorni R. Adikaram, Claire Kittock, Nicole Lue, Adam Awe, Katherine Degner, Nirmal Jacob, Jenna Staples, Rachel Thomas, Allison B. Kohnen, Sundar Ganesan, Juraj Kabat, Ching-Kang Chen, William F. Simonds

**Affiliations:** 1 Metabolic Diseases Branch, National Institute of Diabetes and Digestive and Kidney Diseases, National Institutes of Health (NIH), Bethesda, Maryland, USA.; 2 Research Technologies Branch, National Institute of Allergy and Infectious Diseases, NIH, Bethesda, Maryland, USA.; 3 Department of Molecular Medicine, The University of Texas Health Science Center at San Antonio, San Antonio, Texas, USA.

**Keywords:** Neuroscience, G protein&ndash;coupled receptors, G proteins, Pain

## Abstract

Mechanical, thermal, and chemical pain sensation is conveyed by primary nociceptors, a subset of sensory afferent neurons. The intracellular regulation of the primary nociceptive signal is an area of active study. We report here the discovery of a Gβ5-dependent regulatory pathway within mechanical nociceptors that restrains antinociceptive input from metabotropic GABA-B receptors. In mice with conditional knockout (cKO) of the gene that encodes Gβ5 (*Gnb5*) targeted to peripheral sensory neurons, we demonstrate the impairment of mechanical, thermal, and chemical nociception. We further report the specific loss of mechanical nociception in Rgs7-Cre^+/–^ *Gnb5*^fl/fl^ mice but not in Rgs9-Cre^+/–^ *Gnb5*^fl/fl^ mice, suggesting that Gβ5 might specifically regulate mechanical pain in regulator of G protein signaling 7–positive (Rgs7^+^) cells. Additionally, Gβ5-dependent and Rgs7-associated mechanical nociception is dependent upon GABA-B receptor signaling since both were abolished by treatment with a GABA-B receptor antagonist and since cKO of Gβ5 from sensory cells or from Rgs7^+^ cells potentiated the analgesic effects of GABA-B agonists. Following activation by the G protein–coupled receptor Mrgprd agonist β-alanine, enhanced sensitivity to inhibition by baclofen was observed in primary cultures of Rgs7^+^ sensory neurons harvested from Rgs7-Cre^+/–^ *Gnb5*^fl/fl^ mice. Taken together, these results suggest that the targeted inhibition of Gβ5 function in Rgs7^+^ sensory neurons might provide specific relief for mechanical allodynia, including that contributing to chronic neuropathic pain, without reliance on exogenous opioids.

## Graphical abstract

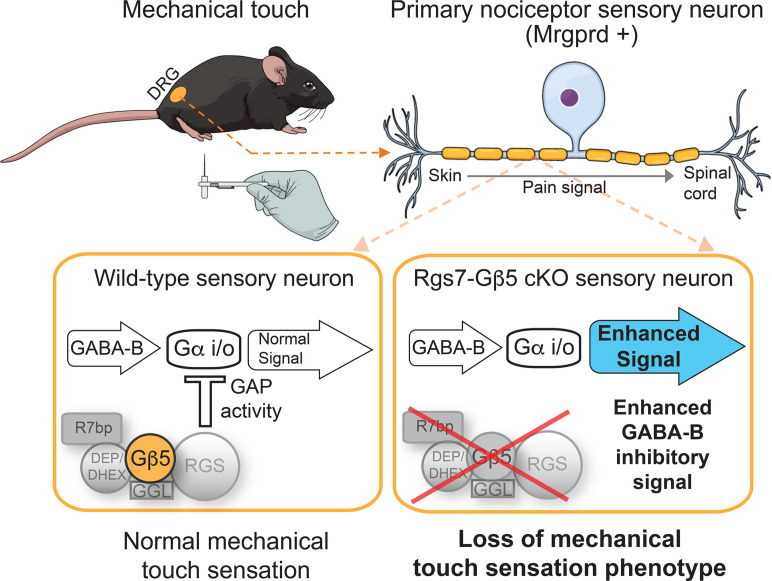

## Introduction

Neuropathic pain not only can result from disorders of the central nervous system, such as stroke and spinal cord injury, but also can be the consequence of peripheral neuropathies that disrupt somatosensation. Painful diabetic polyneuropathy, for example, affects as many as 100 million people worldwide (1, 2).

Opioid derivatives are currently the most widely used painkillers. However, patients with chronic pain, including some with painful neuropathies, are at risk for opioid dependence. The lack of effective alternatives to narcotic analgesics is a contributing factor in the current nationwide opioid crisis, pointing to an urgent public health need to identify alternative drugs that can regulate nociceptive pathways. A relatively unexplored area of research is the targeting of intracellular downstream targets of receptors regulating nociceptor function.

Many types of receptors present on nociceptive sensory neurons have been implicated in ameliorating pain, including G protein–coupled receptors (GPCRs) such as the Gi/o-coupled opioid receptors that activate pertussis toxin–sensitive signaling pathways. Less is known, however, about how pain signals are processed and regulated inside sensory neurons before the final output of the nociceptive signal at the spinal level.

Regulator of G protein signaling (RGS) proteins negatively regulate GPCR signaling in many cell types (3). RGS proteins rapidly terminate G protein signaling by acting as GTPase-activating proteins (GAPs) for G protein α subunits, accelerating the intrinsic hydrolysis rate of Gα-bound GTP. The R7-RGS subfamily of RGS proteins, consisting of RGS6, RGS7, RGS9, and RGS11, share the ability to form obligate heterodimers with Gβ5, thereby stabilizing the Gβ5/R7-RGS complex (4–6). Germline knockout of Gβ5 destabilizes all R7-RGS proteins, leading to their loss of expression (7). A body of in vitro evidence suggests that the GAP activity of Gβ5**/**R7-RGS complexes is specific for pertussis toxin–sensitive Gα subunits (Gαo and/or Gαi) (8–11). Although the physiology of endogenous Gβ5**/**R7-RGS complexes within the central and peripheral nervous system in vivo is poorly understood, the presence of Gβ5 and other components of the heterotrimeric Gβ5**/**R7-RGS complex in sensory neurons (12, 13) suggests that the Gβ5 protein complex might function there in the regulation of somatosensation, including perhaps nociception.

We report here that conditional knockout (cKO) of the gene that encodes Gβ5 (*Gnb5*) from sensory neurons impairs multiple modalities of nociception. Mechanical nociception was specifically impeded by the targeted KO of *Gnb5* from Rgs7^+^ but not Rgs9^+^ neurons. The *Gnb5*-dependent and Rgs7-associated mechanical nociception was dependent on GABA-B signaling, suggesting a model whereby Gβ5, present in Rgs7^+^ primary sensory neurons, normally suppresses a GABA-B receptor–mediated signal that inhibits mechanical pain. Such a model implies that the therapeutic inhibition of Gβ5-dependent GAP activity in Rgs7^+^ sensory neurons might provide specific relief for chronic neuropathic pain, characterized by mechanical allodynia, by amplifying or exaggerating endogenous GABAergic tone.

## Results

### Targeted knockout of Gnb5 from sensory afferents impairs nociception.

**Figure 1:**
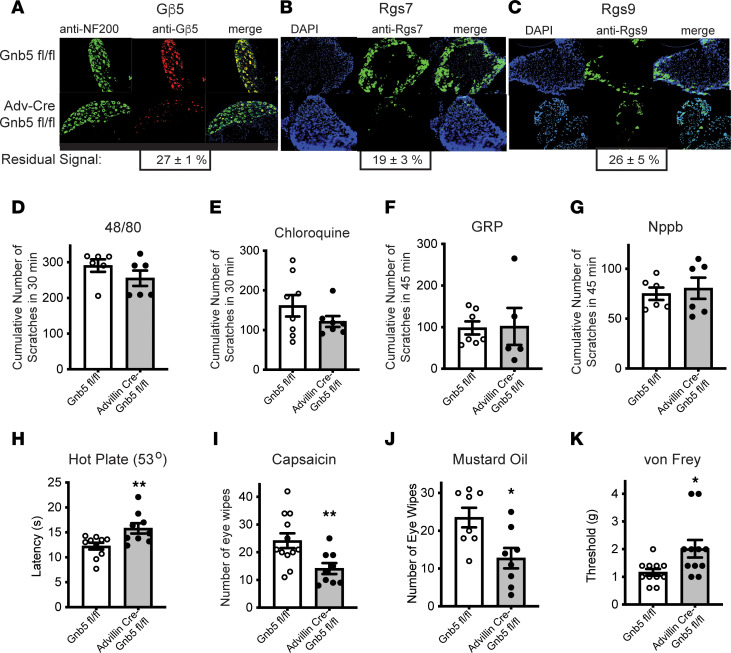
Knockout of *Gnb5* from sensory neurons destabilizes its R7-RGS protein partners and impairs multiple modalities of nociception but not pruriception. (**A**–**C**) Sections through lumbar dorsal root ganglia (DRG) from mice harboring a conditional *Gnb5*-knockout allele (*Gnb5*^fl/fl^) alone (upper panels) or Advillin (Adv)-Cre^+/–^ *Gnb5*^fl/fl^ mice (lower panels). (**A**) DRG analyzed by dual immunohistochemistry (IHC) with antibodies against NF200 (Nefh) (green) or Gβ5 (red) as indicated. (**B** and **C**) DRG analyzed by combined DAPI staining (blue) and IHC with antibodies against Rgs7 (**B**) or Rgs9 (**C**) (green). The percentage of residual antibody signal following Adv-Cre–mediated knockout of *Gnb5* is indicated below each image (mean ± SEM). (**D** and **E**) Cumulative scratching behavior in mice of the indicated genotypes induced by the intradermal administration of (**D**) compound 48/80 or (**E**) chloroquine (64, 65). (**F** and **G**) Cumulative scratching behavior in mice of the indicated genotypes induced by the intrathecal injection of gastrin-releasing peptide (GRP) (**F**) or Nppb (**G**). (**H**–**K**) Behavioral testing of nociception in littermates with either the *Gnb5*^fl/fl^ or Adv-Cre^+/–^ *Gnb5*^fl/fl^ genotypes, as indicated. (**H**) Hot plate testing (53°C). (**I**) Eye wipe testing using capsaicin. (**J**) Eye wipe testing of chemical nociception using mustard oil. (**K**) Von Frey filament behavioral testing of mechanical nociception. *N* values provided in Methods. For **D**–**K**, the genotype of the mouse being tested was masked to the evaluator. For **D**, **E**, and **K**, the Mann-Whitney test was employed, and for **F**–**J**, the 2-tailed unpaired Student’s *t* test was employed, with bars indicating mean ± SEM. *P* values: **D**, *P* = 0.38; **E**, *P* = 0.52; **F**, *P* = 0.92; **G**, *P* = 0.67; **H**, **P = 0.0095; **I**, **P = 0.0098; **J**, *P = 0.012; **K**, *P = 0.02. For **A**–**C**, DRG derived from 5 mice of each genotype were examined, with representative sections shown.

We and others have shown that Gβ5 and other components of the heterotrimeric Gβ5**/**R7-RGS complex are expressed in neurons of the dorsal root ganglia (DRG) (12, 13). To better understand the possible role of the Gβ5**/**R7-RGS complex in sensory neuronal function, we used a Cre/*loxP*-mediated conditional gene knockout strategy targeting *Gnb5* expression in peripheral neurons. To this end, we utilized mice expressing Cre recombinase under the control of the Advillin promoter (Advillin-Cre^+/–^) (14–16). Crossing such Advillin-Cre^+/–^ mice with mice carrying an Ai9-Cre reporter allele (17) resulted in progeny expressing tdTomato fluorescence in peripheral sensory neurons, consistent with previous reports (14–16). We then crossed Advillin-Cre^+/–^ mice with mice harboring a pair of *loxP* sites flanking exon 3 of the *Gnb5* gene (*Gnb5*^fl/fl^). The resulting Advillin-Cre^+/–^ *Gnb5*^fl/fl^ mice showed the expected loss of Gβ5 expression within sensory afferent neurons when DRG were analyzed by immunohistochemistry (IHC) (Figure 1A). Gβ5 forms a tight, noncovalent bond with R7-RGS proteins through their Gγ-like domain (18), such that lack of Gβ5 destabilizes all R7-RGS proteins, leading to their loss of expression (7). DRG harvested from Advillin-Cre^+/–^ *Gnb5*^fl/fl^ mice showed loss of Rgs7 (Figure 1B) and Rgs9 (Figure 1C) expression by IHC, consistent with the destabilization of Gβ5**/**R7-RGS complexes in sensory neurons under these conditions. The ability to selectively knock down Gβ5 and its obligate protein partners from Advillin^+^ cells allowed us to test if the Gβ5 present in sensory neurons might regulate critical modalities of somatosensation. Itch sensation was tested by quantitating scratching behavior following the administration of pruritogens. No significant differences in apparent pruriception between control and Advillin-Cre^+/–^ *Gnb5*^fl/fl^ mice were observed (Figure 1, D–G).

The capsaicin-sensitive transient receptor potential vanilloid 1 (Trpv1) channel expressed in peripheral sensory neurons mediates thermal nociception in vertebrates. Thermal nociception tested using the hot plate test was diminished in the Advillin-Cre^+/–^ *Gnb5*^fl/fl^ mice compared with control as evidenced by enhanced basal latency of jumping and paw licking (Figure 1H). Acute chemical nociception, as determined by the eye wipe test with the Trpv1 agonist capsaicin, was also impaired in Advillin-Cre^+/–^ *Gnb5*^fl/fl^ mice compared with *Gnb5*^fl/fl^ control littermates (Figure 1I).

Acute chemical nociception in response to mustard oil (acting through transient receptor potential ankyrin 1 [Trpa1] cation channels) was impaired in Advillin-Cre^+/–^ *Gnb5*^fl/fl^ mice compared with *Gnb5*^fl/fl^ control littermates as evidenced by a reduction in eye-wiping behavior (Figure 1J). Similarly, mechanical nociception, a sensation that depends on sensory neurons expressing the GPCR Mrgprd in adult mice (19), was diminished in Advillin-Cre^+/–^ *Gnb5*^fl/fl^ mice compared with *Gnb5*^fl/fl^ controls as demonstrated by testing with von Frey filaments (Figure 1K).

**Figure 2:**
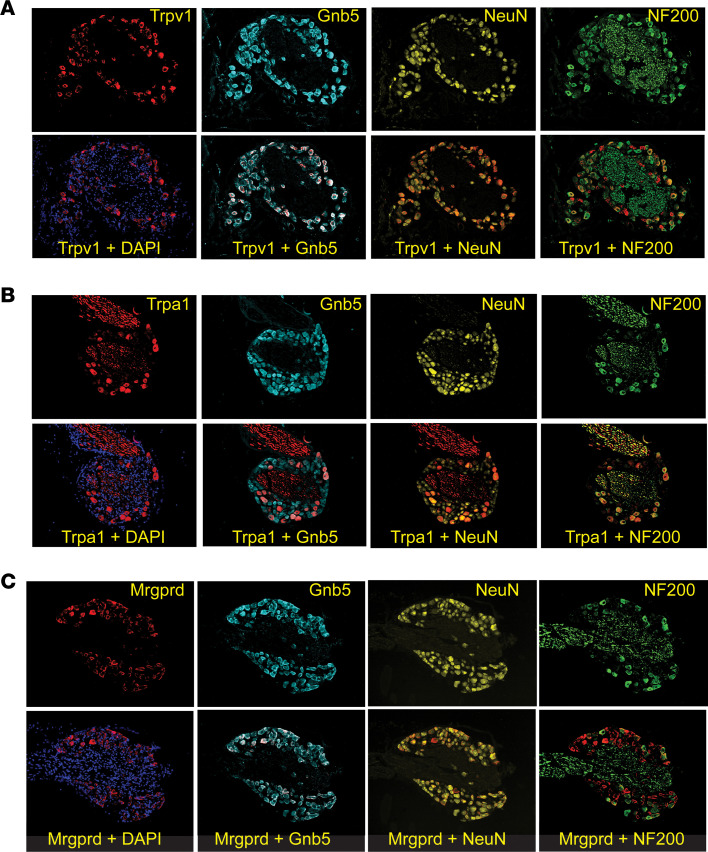
Expression of *Gnb5* and nociceptive markers in sensory neurons. Sections through lumbar dorsal root ganglia (DRG) harvested from wild-type mice and analyzed by immunohistochemistry with antibodies against Trpv1 (**A**), Trpa1 (**B**), or Mrgprd (**C**) (red) and following counterstaining with DAPI (purple) or costaining with additional antibodies against G5 (*Gnb5*) (blue), NeuN (gold), or NF200 (Nefh) (green) as indicated and as described in Methods. DRG from 4 to 5 mice of each genotype were examined, with representative sections shown.

One explanation for the above findings could be the existence of a regulatory role for Gβ5 in nociception at the primary neuronal level. Coexpression of Gβ5 in the same sensory neurons that also express molecules mediating the various nociceptive stimuli might support such a model. To explore this possibility, we surveyed DRG for the expression of Gβ5, Trpv1, Trpa1, Mrgprd, and other neuronal markers at the protein level in wild-type mice by IHC. As seen in Figure 2A, Trpv1 antibodies labeled a subset of the sensory ganglionic neurons that could be identified by counterstaining with 4′,6-diamidino-2-phenylindole (DAPI) or with antibody against NeuN, a marker present in most vertebrate neurons. Many of the Gβ5-positive cells also expressed Trpv1 while the majority of Trpv1-positive cells coexpressed Gβ5. Only a fraction of cells positive for antibody against neurofilament 200 (NF200; aka neurofilament heavy chain, Nefh) also expressed Trpv1, consistent with transcriptomic profiling of mouse sensory neurons by single-cell RNA-Seq (20). Most Trpa1-positive cells also expressed Gβ5, whereas antibodies against Trpa1 labeled only a subset of the Gβ5-positive cells (Figure 2B). With respect to the expression of Mrgprd (Figure 2C), antibodies against Mrgprd labeled a small fraction of the total Gβ5-positive cells, yet nearly all Mrgprd-positive cells also expressed Gβ5. Also consistent with prior transcriptomic sensory neuron profiling, almost no cells labeled with antibody against Mrgprd also expressed NF200 (20). Taken together, the demonstration that most sensory ganglionic cells expressing Trpv1, Trpa1, and Mrgprd coexpress Gβ5 would be consistent with a regulatory role for the latter in nociception at the primary neuronal level.

### Gβ5’s presence in Rgs9-expressing neurons is required for thermal sensitivity.

Given Gβ5’s canonical role in the stabilization of Gβ5**/**R7-RGS complexes (7), the diminution of thermal, chemical, and mechanical nociception observed in Advillin-Cre^+/–^ *Gnb5*^fl/fl^ mice suggested that the phenotypes probably involve regulation by 1 or more of the 4 R7 subfamily RGS proteins, namely RGS6, -7, -9, or -11. To further study this question, we used mice expressing Cre recombinase under the control of the endogenous Rgs7 or Rgs9 (21) promoters (Rgs7-Cre^+/–^ and Rgs9-Cre^+/–^, respectively). Neither Rgs7-Cre^+/–^ nor Rgs9-Cre^+/–^ mice exhibited a thermal, chemical, or mechanical nociceptive phenotype distinct from the wild-type (Supplemental Figure 1; supplemental material available online with this article; https://doi.org/10.1172/jci.insight.134685DS1). Since Rgs9 has been previously implicated in nociceptive behavior (22), we first looked for possible Gβ5-dependent nociceptive pathways in Rgs9-expressing neurons by utilizing mice expressing Cre recombinase under the control of the Rgs9 promoter (21).

The validity of the transgenic Rgs9-Cre construct in sensory neurons was confirmed by crossing Rgs9-Cre^+/–^ mice with Ai9 reporter mice expressing tdTomato in a Cre-dependent fashion (17). DRG harvested from Rgs9-Cre^+/–^ Ai9 mice demonstrated a strong tdTomato signal in a subset of sensory neurons (Supplemental Figure 2A). Analysis by IHC and quantification of the same DRG sections provided information about the colocalization of Rgs9 with Gβ5. Costaining of DRG sections with antibodies against Rgs9 suggested the fidelity of the tdTomato signal in Rgs9-Cre^+/–^ Ai9 mice, with nearly 70% colocalization (69.62%, *n* = 4). Analysis of sections with anti-Gβ5 antibodies suggested that fewer than half of Gβ5^+^ cells expressed Rgs9 (39.4%, *n* = 4) (Supplemental Figure 2A).

**Figure 3:**
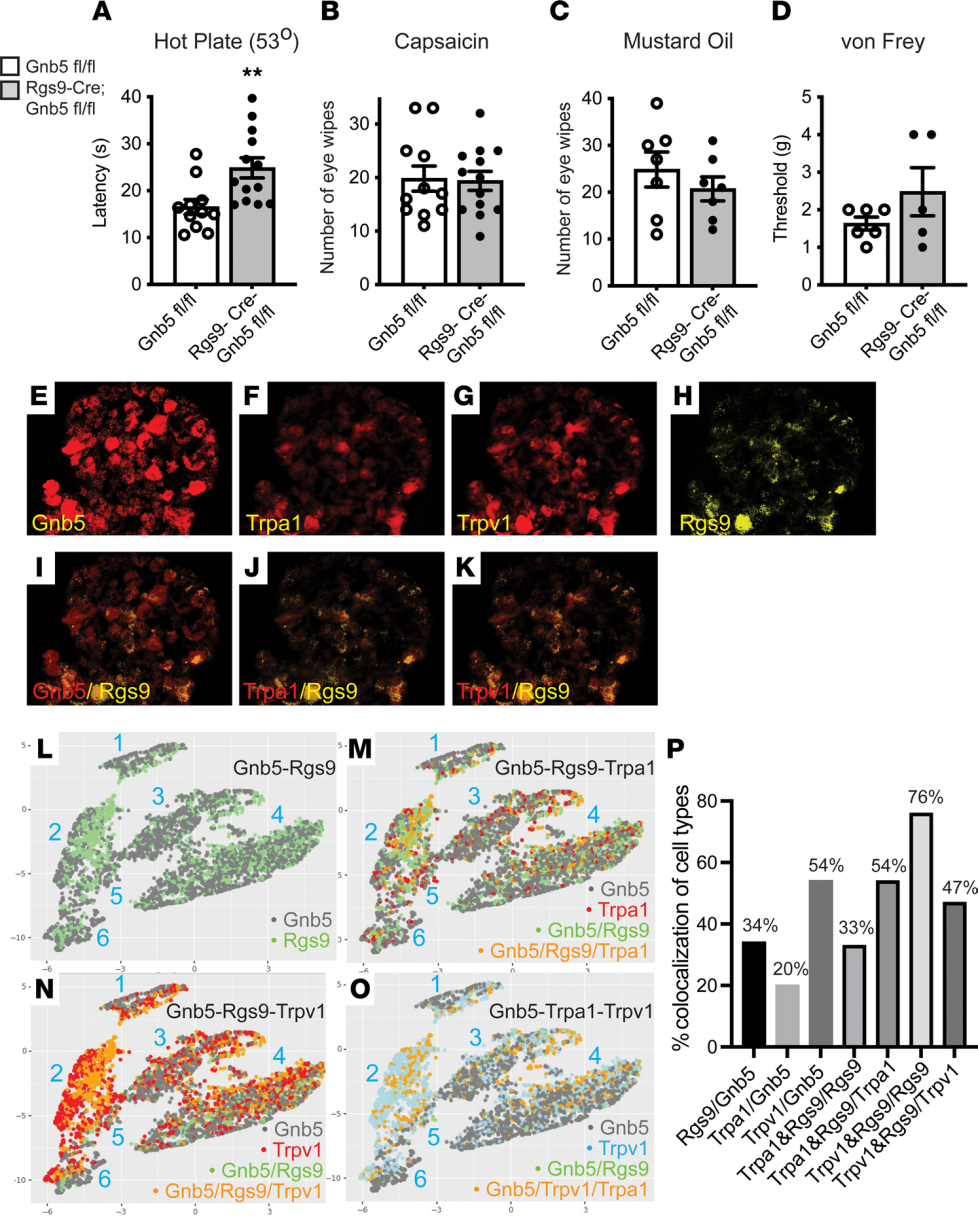
Knockout of *Gnb5* from Rgs9^+^ neurons preserves chemical and mechanical nociception, as well as expression of Rgs9, *Gnb5*, and the nociceptors Trpv1 and Trpa1 in sensory neurons. (**A**–**D**) Behavioral testing of nociception in littermates with either the *Gnb5*^fl/fl^ or else Rgs9-Cre^+/–^ *Gnb5*^fl/fl^ genotypes, as indicated. (**A**) Hot plate testing (53°C). (**B**) Eye wipe testing with capsaicin. (**C**) Eye wipe testing of chemical nociception with mustard oil. (**D**) Von Frey filament behavioral testing of mechanical nociception. For **A**–**D**, the genotype of the mouse being tested was masked. (**E**–**K**) Representative images from RNAscope analysis of transcripts for genes shown (**E**–**H**): Gnb5, Trpa1, and Trpv1 are in red; Rgs9 is in yellow. The colocalization of Rgs9 transcript with that of Gnb5, Trpa1, and Trpv1 is shown (**I**–**K**). (**L**–**O**) Clustering analysis and UMAP visualization of 5,200 DRG expressing 1 or more of 4 gene transcripts above obtained from RNAscope experiments. The text colors in the lower right part of each figure correspond to individual cell coloring, indicating expression of that transcript, or combination of transcripts, in that cell. (**P**) Histogram illustrating the percentages of colocalized genes as shown. In **A**–**D**, the 2-tailed unpaired Student’s *t* test of statistical significance was employed, with bars indicating mean ± SEM. For panels **A**–**D**, number of mice from each genotype tested (for **A**, *Gnb5*^fl/fl^ *n* = 11, Rgs9-Cre^+/–^; *Gnb5*^fl/fl^ *n* = 13; for **B**, *Gnb5*^fl/fl^ *n* = 11, Rgs9-Cre^+/–^; *Gnb5*^fl/fl^ *n* = 13; for **C**, *Gnb5*^fl/fl^ *n* = 7, Rgs9-Cre^+/–^; *Gnb5*^fl/fl^ *n* = 7; for **D**, *Gnb5*^fl/fl^ *n* = 6, Rgs9-Cre^+/–^; *Gnb5*^fl/fl^ *n* = 5). *P* values: **A**, **P = 0.006; **B**, *P* = 0.88; **C**, *P* = 0.38; **D**, *P* = 0.20.

Behavioral assays of nociception in Rgs9-Cre^+/–^ *Gnb5*^fl/fl^ mice were performed, to explore the extent to which Rgs9-expressing neurons might be involved in the nociceptive phenotypes observed in Advillin-Cre^+/–^ *Gnb5*^fl/fl^ mice. Thermal nociception using the hot plate test was significantly diminished in the Rgs9-Cre^+/–^ *Gnb5*^fl/fl^ mice compared with control as evidenced by enhanced basal latency of jumping and paw licking (Figure 3A), consistent with previous studies on Rgs9–global KO mice (23). In contrast, testing for chemical nociception, by capsaicin and mustard oil eye wipe assay, as well as testing for mechanical nociception showed no changes in Rgs9-Cre^+/–^ *Gnb5*^fl/fl^ mice versus control (Figure 3, B–D).

The sensory neurons expressing Rgs9 were profiled in greater depth by analyzing gene expression in 5,200 individual wild-type DRG neurons by in situ hybridization using RNAscope technology (Figure 3, E–O; summarized in Figure 3P). Such analysis revealed that 34% of Gβ5^+^ cells expressed Rgs9, consistent with the IHC results employing anti-Rgs9 antibody above. Considering all Rgs9^+^ sensory neurons, 76% coexpressed Trpv1 while only 33% coexpressed Trpa1 (Figure 3, E–K and P). Knockout of Trpv1 (24), but not of Trpa1 (25), has been shown to interfere with thermal nociception as evidenced by increased latency to nocifensive behavior during 52°C and 55°C hot plate testing. It is therefore plausible that the increased latency observed during 53°C hot plate testing we observed in mice lacking Gβ5 in Rgs9^+^ neurons could be explained, at least in part, by dysfunction of Rgs9^+^Trpv1^+^ sensory neurons.

Application of the Uniform Manifold Approximation and Projection for Dimension Reduction (UMAP) algorithm (26) to the results of the RNAscope in situ hybridization analysis of DRG enabled the assignment of Gβ5^+^ neurons into 6 distinct clusters (clusters 1–6 in Figure 3, L–O). Although Rgs9 transcript was represented among all 6 clusters of Gβ5^+^ cells, Rgs9 expression appeared more concentrated in cluster 2 (Figure 3L). Trpv1^+^ neurons also appeared more highly represented in cluster 2 (Figure 3N), while Trpa1^+^ neurons were distributed more evenly among the 6 clusters (Figure 3M). Loss of Gβ5 from the Rgs9^+^ and Trpv1^+^ neurons, including those concentrated in cluster 2, could reasonably contribute to the altered responses to noxious heat stimuli seen in Rgs9-Cre^+/–^ *Gnb5*^fl/fl^ mice compared with control.

The observation that the targeted KO of *Gnb5* from Rgs9-expressing cells could diminish thermal nociception without impairing capsaicin-mediated chemical nociception in the eye wipe test was unexpected and will be discussed below.

### Gβ5’s presence in Rgs7-expressing neurons is required for mechanical sensitivity.

**Figure 4:**
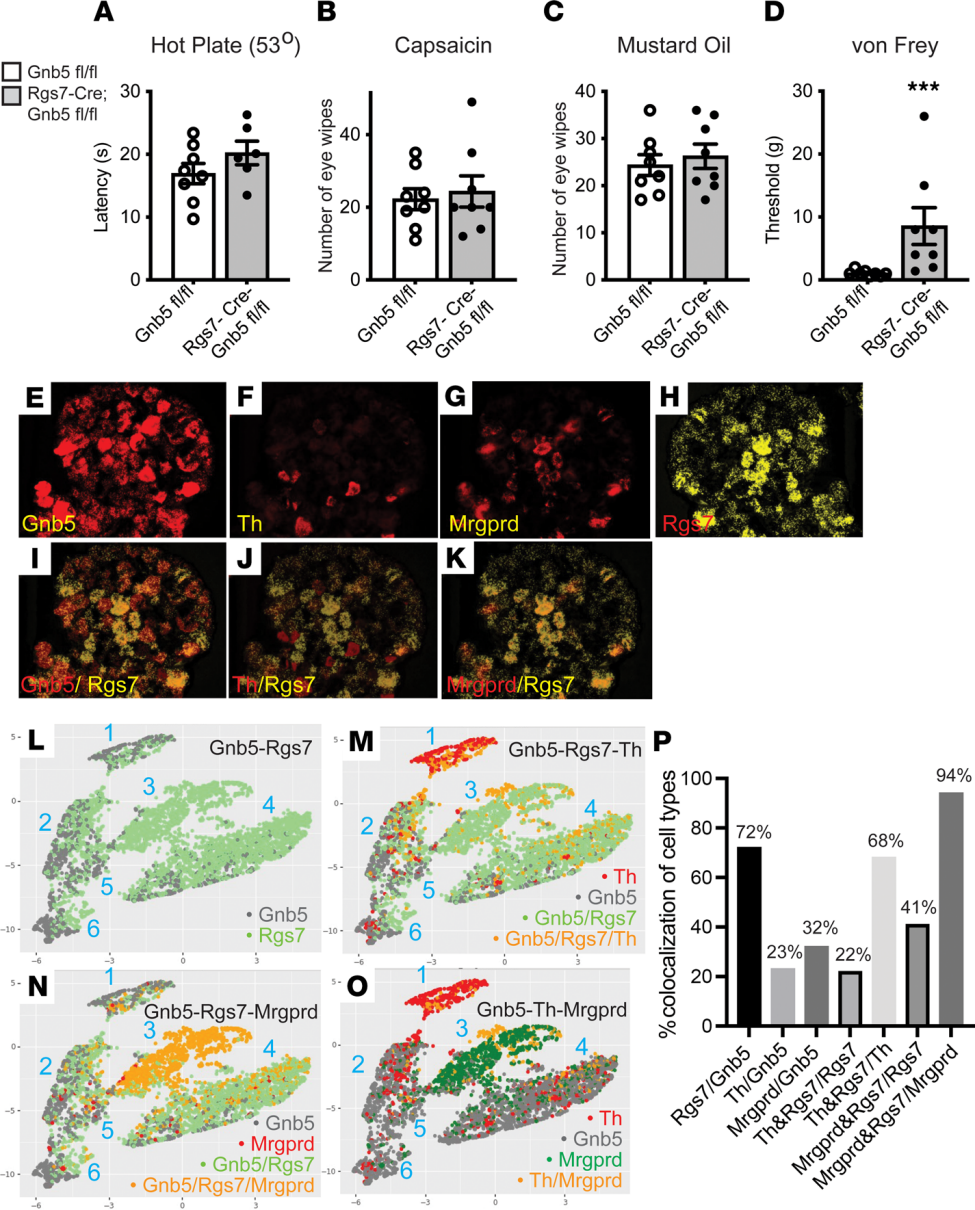
Knockout of *Gnb5* from Rgs7^+^ neurons specifically impairs mechanical nociception and expression of Rgs7, *Gnb5*, Mrgprd, and Th in sensory neurons. (**A**–**D**) Behavioral testing of nociception in littermates with either the *Gnb5*^fl/fl^ or Rgs7-Cre^+/–^ *Gnb5*^fl/fl^ genotypes, as indicated. (**A**) Hot plate testing (53**°**C). (**B**) Eye wipe testing with the Trpv1 agonist capsaicin. (**C**) Eye wipe testing of chemical nociception with mustard oil. (**D**) Von Frey filament behavioral testing of mechanical nociception. (**E**–**K**) Representative images of RNAscope analysis of transcripts for genes shown (**E**–**H**): Gnb5, Th, and Mrgprd are in red; Rgs7 is in yellow. The colocalization of Rgs7 mRNA with that of Gnb5, Th, and Mrgprd is shown (**I**–**K**). (**L**–**O**) Clustering analysis and UMAP visualization of 5,200 DRG expressing 1 or more of the 4 genes above obtained from RNAscope experiments. The text colors in the lower right part of each figure correspond to individual cell coloring, indicating expression of that transcript, or combination of transcripts, in that cell. (**P**) Histogram illustrating the percentages of colocalized genes as shown. For **A**–**D**, the genotype of the mouse being tested was masked. For **A**–**C**, the 2-tailed unpaired Student’s *t* test of statistical significance was employed, and for **D** the Mann-Whitney test was employed, with bars indicating mean ± SEM. For **A**–**D**, *n* = number of mice from each genotype tested (for **A**, *Gnb5*^fl/fl^ *n* = 8, Rgs7-Cre^+/–^; *Gnb5*^fl/fl^ *n* = 6; for **B**, *Gnb5*^fl/fl^ *n* = 8, Rgs7-Cre^+/–^; *Gnb5*^fl/fl^ *n* = 8; for **C**, *Gnb5*^fl/fl^ *n* = 8, Rgs7-Cre^+/–^; *Gnb5*^fl/fl^ *n* = 8; for **D**, *Gnb5*^fl/fl^ *n* = 8, Rgs7-Cre^+/–^; *Gnb5*^fl/fl^ *n* = 8). *P* values: **A**, *P* = 0.69; **B**, *P* = 0.21; **C**, *P* = 0.59; **D**, ***P = 0.0008.

We next looked for possible Gβ5-dependent nociceptive pathways involving Rgs7-expressing sensory neurons. Behavioral assays of nociception were performed in Rgs7-Cre^+/–^ *Gnb5*^fl/fl^ mice, to better understand the extent to which the nociceptive phenotypes observed in Advillin-Cre^+/–^ *Gnb5*^fl/fl^ mice might involve Rgs7-expressing neurons (Figure 4, A–D). While behavioral testing for thermal and chemical nociception showed no changes versus control in Rgs7-Cre^+/–^ *Gnb5*^fl/fl^ mice (Figure 4, A–C), mechanical nociception, as assessed by von Frey filament testing, was diminished as evidenced by a higher mechanical threshold for hind paw withdrawal (Figure 4D).

To validate the transgenic Rgs7-Cre mouse line, we crossed Rgs7-Cre^+/–^ mice with Ai9 reporter mice expressing tdTomato in a Cre-dependent fashion (17). Histofluorescence analysis of DRG harvested from Rgs7-Cre^+/–^ Ai9 mice identified a strong tdTomato signal in a subset of sensory neurons (Supplemental Figure 2B). Costaining of DRG sections with antibodies against Rgs7 validated the fidelity of the tdTomato signal in Rgs7-Cre^+/–^ Ai9 mice, with more than 90% colocalization (94.17%, *n* = 5) (Supplemental Figure 2B).

The sensory neurons expressing Rgs7 were further characterized by in situ hybridization using the RNAscope technology. Profiling analysis of transcripts expressed in 5,200 DRG demonstrated that among Rgs7^+^ neurons, 22% expressed tyrosine hydroxylase (Th) and 41% expressed Mrgprd (Figure 4, E–K and P). Interestingly, 68% of Th^+^ and 94% of Mrgprd^+^ neurons, respectively, coexpressed Rgs7 transcript (Figure 4, E–K and P). Sensory neurons that express Th represent a distinct population of mainly calcitonin gene-related peptide^–^ and isolectin B4^–^ (IB4^–^) afferents (27) that have been implicated in nonvisceral pain sensation (28). It is known that Mrgprd-expressing sensory neurons are required for the full expression of mechanical pain in adult mice (19). These results correlate Rgs7 gene expression with known markers of nociceptive neurons in the DRG.

Analysis of the Gβ5^+^ DRG by the UMAP algorithm revealed different distribution patterns of Rgs7 transcript among the 6 clusters of Gβ5^+^ neurons (clusters 1–6; Figure 4, L–O). Rgs7 transcript was present in all 6 clusters of Gβ5^+^ cells; however, it appeared most concentrated in clusters 3 and 4 (Figure 4, L–O). The Th^+^ neurons were primarily associated with cluster 1 (Figure 4, M and O) while most of the Mrgprd^+^ neurons were concentrated in cluster 3 (Figure 4, N and O), suggesting that the Th and Mrgprd genes function in distinct subsets of cells. It is noteworthy that transcriptomic profiling of mouse sensory neurons by single-cell RNA-Seq also found Th and Mrgprd to be markers of distinct categories of sensory cells (20). This finding is further supported by our results showing that very few cells express both Th and Mrgprd transcripts (Figure 4O). These results imply that although Th-expressing and Mrgprd-expressing neurons may represent distinct subsets of nociceptors (cluster 1 and cluster 3, respectively), the functions of both neuronal subsets may be regulated by Gβ5, and possibly Rgs7, gene expression.

### Rgs7-associated and Gβ5-dependent mechanical nociception depends on GABA-B receptor signaling.

As previously noted, mechanical allodynia in adult mice as assessed by von Frey filament testing depends on sensory neurons that express the GPCR Mrgprd (19). The large overlap between Mrgprd and Rgs7 expression evident by in situ hybridization in DRG sensory neurons (Figure 4, N and P) suggests a molecular mechanism for the significant loss in mechanical nociception observed when *Gnb5* KO was targeted to Rgs7^+^ neurons (Figure 4D). We hypothesized that a functional Gβ5/R7-RGS complex expressed in Rgs7^+^ and Mrgprd^+^ sensory cells could regulate mechanical nociception. If the GAP activity of a Gβ5/R7-RGS complex in these cells normally acted to restrain inhibitory, i.e., antinociceptive, signaling by a GPCR expressed on the surface of Mrgprd^+^ afferents, then knockout of Gβ5 and loss of such GAP activity would enhance or exaggerate this antinociceptive GPCR signal, resulting in a possible loss-of-nociception phenotype. We therefore considered candidate inhibitory GPCRs.

The GABA-B receptor is a metabotropic Gi/o-coupled receptor for GABA previously implicated in the regulation and perception of pain in the central nervous system (29, 30). Upon agonist binding, the GABA-B receptor typically inhibits neurons via activation of G protein–coupled inwardly rectifying potassium (K^+^) (GIRK) channels and inhibition of N-type and P/Q-type members of the voltage-gated calcium channel family. Both types of neuronal inhibition are mediated by G heterodimers released from Gi/o (31, 32), and both have been previously shown to be regulated by Gβ5/Rgs7 complexes (33–35). Activation of GIRK channels by GABA-B receptor agonists has also been reported in DRG neurons (36, 37). To test the possible involvement of GABA-B receptors in the loss-of-nociception phenotypes observed following targeted knockout of Gβ5, we employed 2-hydroxysaclofen, a selective GABA-B receptor antagonist.

**Figure 5:**
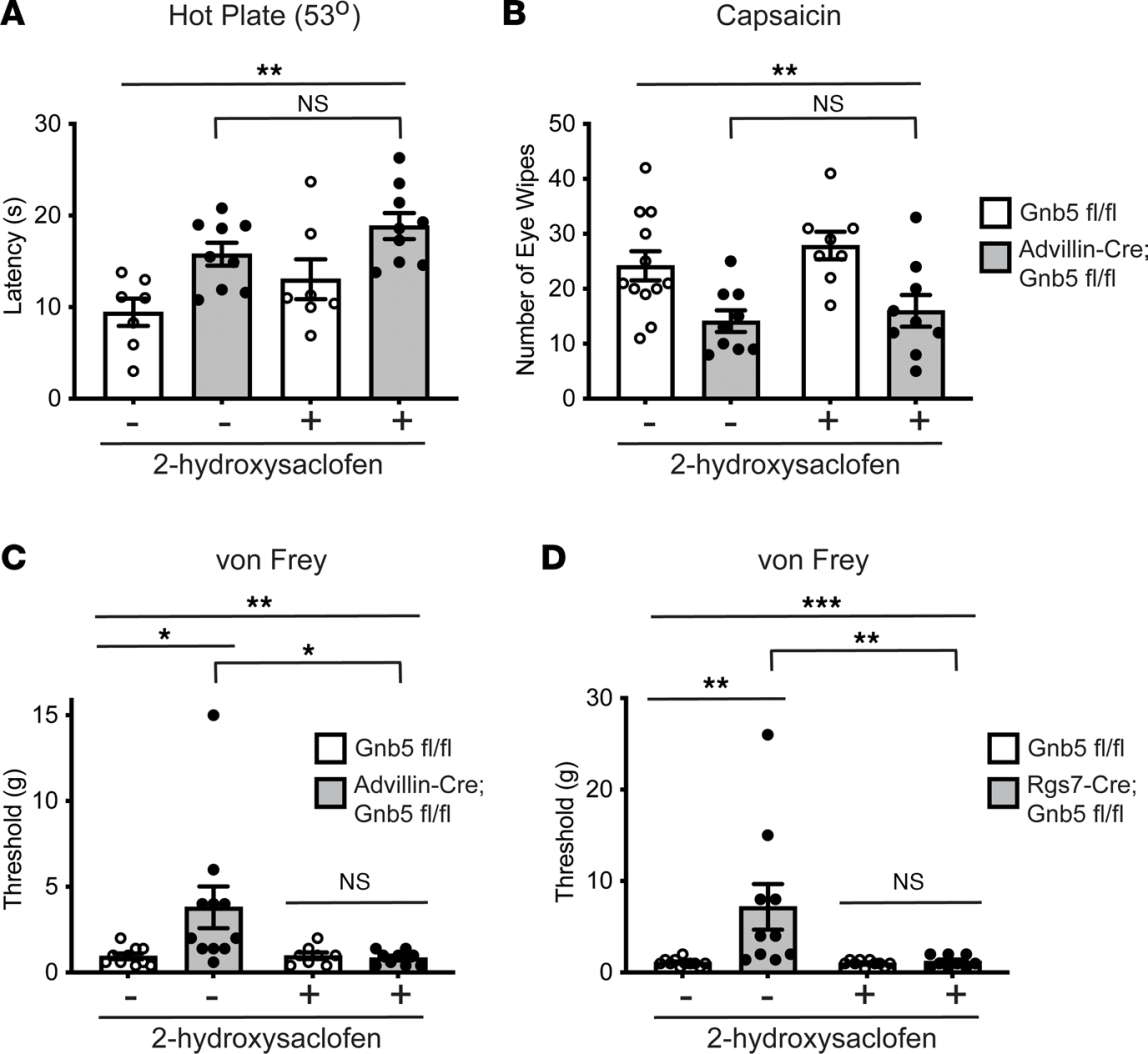
*Gnb5*-dependent and Rgs7-related regulation of mechanical nociception in sensory neurons is abolished by GABA-B receptor antagonism. (**A**–**C**) Behavioral testing of nociception in littermates with *Gnb5*^fl/fl^ or Advillin-Cre^+/–^ *Gnb5*^fl/fl^ genotypes, as indicated, without and with the systemic administration of the GABA-B receptor antagonist 2-hydroxysaclofen (2HS). (**A**) Hot plate testing (53°C). (**B**) Eye wipe testing with capsaicin. (**C**) Von Frey filament testing of mechanical nociception. (**D**) Von Frey filament testing of mechanical nociception in *Gnb5*^fl/fl^ or Rgs7-Cre^+/–^ *Gnb5*^fl/fl^ littermate mice, as indicated, without and with the systemic administration of 2HS. *N* values provided in Methods. For **A**–**D**, the evaluator was masked to the genotype of the mouse being tested and to drug treatment. For **A**, **C**, and **D**, the Kruskal-Wallis (KW) test with Dunn’s multiple comparisons (Dunn’s) testing was employed, and for **B**, 1-way ANOVA with Tukey’s multiple comparisons testing was employed, with bars indicating mean ± SEM. *P* values: **A**, KW test **P = 0.004; **A**, Advillin-Cre^+/–^ *Gnb5*^fl/fl^ without versus with 2HS, Dunn’s test NS *P* > 0.99; **B**, 1-way ANOVA **P = 0.002; **B**, Advillin-Cre^+/–^ *Gnb5*^fl/fl^ without versus with 2HS, Tukey’s test NS *P* = 0.957; **C**, KW test **P = 0.0076; **C** control Dunn’s test *P = 0.02, **C** 2HS Dunn’s test NS *P* > 0.99, **C** Advillin-Cre^+/–^ *Gnb5*^fl/fl^ without versus with 2HS, Dunn’s test *P = 0.021; **D**, KW test ***P = 0.0004; **D** control Dunn’s test **P = 0.002, **D** 2HS Dunn’s test NS *P* > 0.99; **D**, Rgs7-Cre^+/–^ *Gnb5*^fl/fl^ without versus with 2HS, Dunn’s test **P = 0.007.

Administration of 2-hydroxysaclofen had no effect on thermal nociception using the hot plate test or on the eye wipe test with the Trpv1 agonist capsaicin in Advillin-Cre^+/–^ *Gnb5*^fl/fl^ mice compared to *Gnb5*^fl/fl^ control littermates (Figure 5, A and B). Diminished mechanical nociception was observed in Advillin-Cre^+/–^ *Gnb5*^fl/fl^ mice versus control, as demonstrated by von Frey testing (cf. Figure 1K), but that difference was eliminated following treatment with the GABA-B receptor antagonist (Figure 5C).

Given the high degree of Mrgprd and Rgs7 coexpression in sensory afferents and the significant loss in mechanical nociception observed when *Gnb5* KO was targeted to Rgs7^+^ neurons (Figure 4, D and N), we also administered 2-hydroxysaclofen to Rgs7-Cre^+/–^ *Gnb5*^fl/fl^ mice and performed behavioral testing. Treatment with 2-hydroxysaclofen completely abolished the loss of mechanical nociception in Rgs7-Cre^+/–^ *Gnb5*^fl/fl^ mice (Figure 5D). These results, taken together with those of Figure 1K and Figure 4D, suggest that Gβ5-dependent and Rgs7-associated regulation of mechanical allodynia in sensory neurons is dependent on GABA-B receptor signaling.

### Knockout of Gβ5 from sensory or Rgs7-expressing neurons sensitizes mice to the analgesic effects of GABA-B receptor agonists.

To further test the possible involvement of the GABA-B receptor in the nociceptive phenotypes observed following targeted knockout of Gβ5, we utilized a subacute inflammatory pain model induced by intraplantar injection of complete Freund’s adjuvant (CFA) into the mouse hind paw (38). Mechanical nociception was assessed before and 24 hours following the paw injection, without and with the administration of baclofen, a GABA-B receptor agonist.

**Figure 6:**
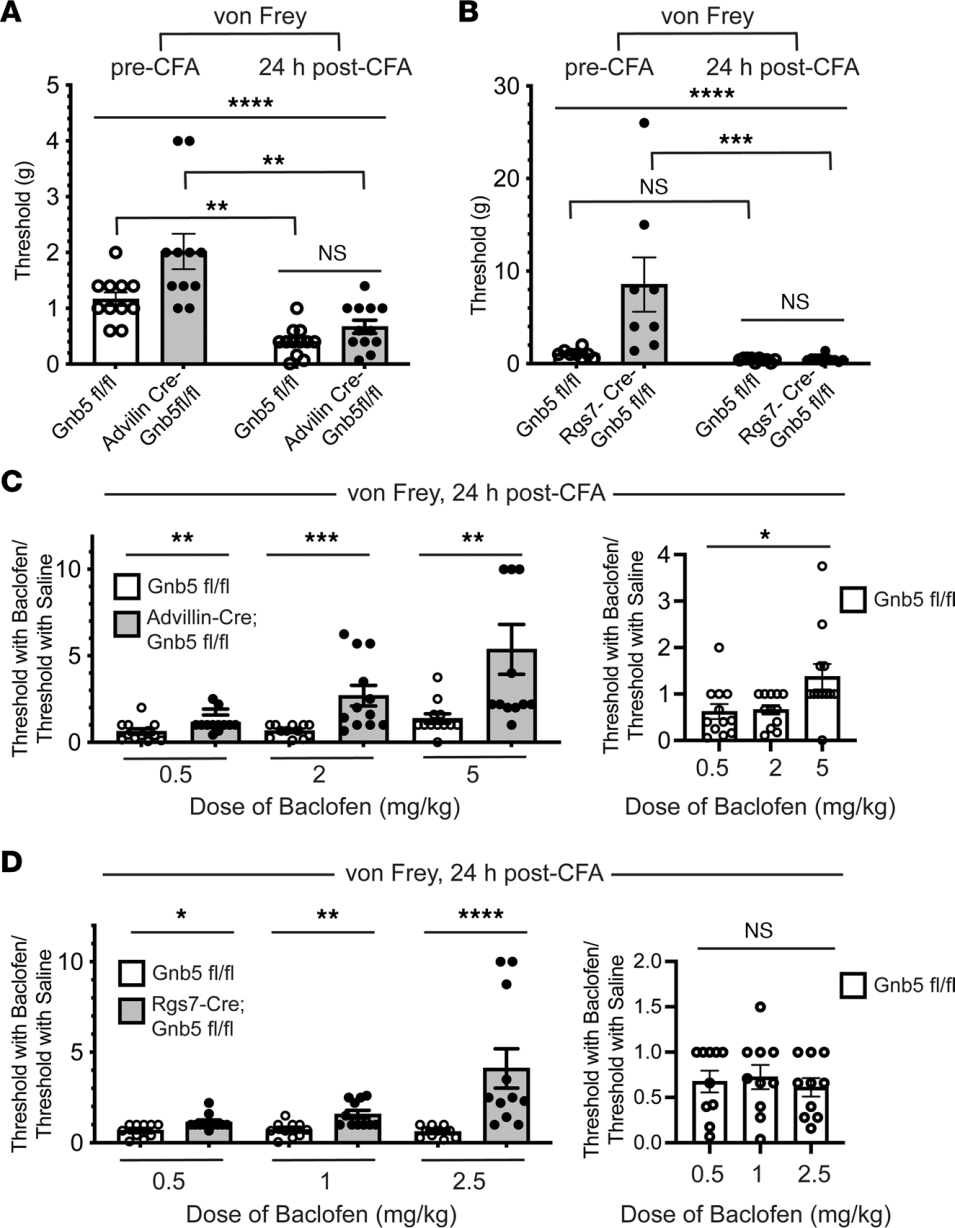
Analgesic effects of GABA-B agonist potentiated by loss of *Gnb5* from sensory cells and from Rgs7^+^ cells in a model of subacute inflammatory pain. (**A** and **B**) Mechanical nociception before and 24 hours following (24hf) the intraplantar injection of complete Freund’s adjuvant (CFA). (**A**) Control and Advillin (Adv)-Cre^+/–^ *Gnb5*^fl/fl^ littermates tested before and after CFA. (**B**) Control and Rgs7-Cre^+/–^ *Gnb5*^fl/fl^ littermates tested before and after CFA. (**C**) (Left) Control and Adv-Cre^+/–^ *Gnb5*^fl/fl^ littermates tested 24hf CFA and 30 minutes following (30mf) the systemic administration of baclofen. (Right) Comparison of control *Gnb5*^fl/fl^ mice only. (**D**) (Left) Control and Rgs7-Cre^+/–^ *Gnb5*^fl/fl^ littermates tested 24hf CFA and 30mf baclofen. (Right) Comparison of control *Gnb5*^fl/fl^ mice only. *N* values provided in Methods. For **A**–**D**, evaluator was masked to both genotype and drug treatment. For **A** and **B**, Kruskal-Wallis (KW) testing with Dunn’s multiple comparisons testing (Dunn’s) employed, with bars indicating mean ± SEM. For **C** and **D** (left), Mann-Whitney test was employed. For **C** and **D** (right), KW testing was employed. *P* values: **A**, KW ****P < 0.0001, **A** *Gnb5*^fl/fl^ mice pre- vs. post-CFA, Dunn’s **P = 0.0045, **A** Adv-Cre^+/–^ *Gnb5*^fl/fl^ mice pre- vs. post-CFA, Dunn’s **P = 0.0014, **A** *Gnb5*^fl/fl^ vs. Adv-Cre^+/–^ *Gnb5*^fl/fl^ mice post-CFA Dunn’s NS; **B**, KW ****P < 0.0001, **B** *Gnb5*^fl/fl^ mice pre- vs. post-CFA, Dunn’s NS, **B** Rgs7-Cre^+/–^ *Gnb5*^fl/fl^ mice pre- vs. post-CFA, Dunn’s ***P = 0.0002, **B** *Gnb5*^fl/fl^ vs. Rgs7-Cre^+/–^ *Gnb5*^fl/fl^ post-CFA Dunn’s NS; **C** left, 0.5 mg/kg dose **P = 0.009; **C** left, 2 mg/kg dose ***P = 0.0003; **C** left, 5 mg/kg dose **P = 0.001; **C** right, *P = 0.01; **D** left, 0.5 mg/kg dose *P = 0.01; **D** left, 1 mg/kg dose **P = 0.003; **D** left, 2.5 mg/kg dose ****P < 0.0001; **D** right, NS.

In both *Gnb5*^fl/fl^ control and Advillin-Cre^+/–^ *Gnb5*^fl/fl^ mice, the induction of mechanical hyperalgesia was evident when thresholds pre- and post-CFA were compared within genotypes using von Frey filaments (Figure 6A). Diminished mechanical nociception in Advillin-Cre^+/–^ *Gnb5*^fl/fl^ mice compared with *Gnb5*^fl/fl^ controls was not demonstrable post-CFA (Figure 6A). Administration of the GABA-B receptor agonist baclofen increased the threshold for mechanical hyperalgesia in Advillin-Cre^+/–^ *Gnb5*^fl/fl^ mice, compared with *Gnb5*^fl/fl^ control littermates, in a dose-dependent fashion, as demonstrated by testing with von Frey filaments (Figure 6C). In addition, at the highest dose of baclofen, the thresholds in the *Gnb5*^fl/fl^ control mice were also significantly increased (Figure 6C).

Increased sensitivity to mechanical stimulation with von Frey filaments was also evident in Rgs7-Cre^+/–^ *Gnb5*^fl/fl^ mice when mechanical thresholds pre- and post-CFA were compared (Figure 6B). Following CFA treatment no significant difference in mechanical nociception between the *Gnb5*^fl/fl^ and Rgs7-Cre^+/–^ *Gnb5*^fl/fl^ genotypes was demonstrable (Figure 6B).

While testing of post-CFA controls and Advillin-Cre^+/–^ *Gnb5*^fl/fl^ mice utilized doses of baclofen between 0.5 and 5 mg/kg (Figure 6C), it was necessary to employ a lower range of baclofen doses in experiments involving Rgs7-Cre^+/–^ *Gnb5*^fl/fl^ mice (0.5 to 2.5 mg/kg) due to sedative effects we observed with higher baclofen doses. Similar sedation and impaired locomotor activity in response to 5 and 10 mg/kg doses of baclofen were previously reported in *Gnb5*^–/–^ mice (39).

Similar to what was observed in the Advillin-Cre^+/–^ *Gnb5*^fl/fl^ mice, testing with von Frey filaments demonstrated that the GABA-B receptor agonist baclofen significantly increased the threshold for mechanical hyperalgesia in Rgs7-Cre^+/–^ *Gnb5*^fl/fl^ mice, compared with *Gnb5*^fl/fl^ control littermates, in a dose-dependent fashion (Figure 6D). None of the baclofen doses, up to 2.5 mg/kg, affected the mechanical thresholds in the *Gnb5*^fl/fl^ control mice in these experiments (Figure 6D).

### Knockout of Gβ5 enhances sensitivity to baclofen in primary cultures of Rgs7^+^ sensory neurons following activation of the Mrgprd receptor.

The above experiments implicate GABA-B signaling in the loss-of-nociception phenotype observed in mice lacking Gβ5 in sensory neurons and in Rgs7^+^ neurons but do not answer the question of whether the GABA-B receptor–mediated and Gβ5-dependent effects are cell autonomous in Rgs7^+^ sensory neurons. That is to say, the above experiments do not link Rgs7, Gβ5, and the GABA-B receptor all together within the same primary mechanical nociceptors and do not exclude a model in which the influence of GABA-B receptors results from nonautonomous crosstalk between different neurons.

To address this issue, functional studies were performed in primary cultures of sensory neurons derived from mouse DRG and loaded with the green-fluorescence calcium indicator, Fluo-4. The sensory neurons were isolated from DRG harvested from mice with Rgs7-Cre^+/–^ and Rgs7-Cre^+/–^ *Gnb5*^fl/fl^ genotypes, established in primary culture, and then transduced with a recombinant adeno-associated virus (rAAV) construct designed to express tdTomato in a Cre-dependent fashion. Introduction of the Cre-dependent tdTomato allele allowed the selection of Rgs7^+^ neurons for functional study, amid a large background population of primary sensory neurons with diverse and unknown functions and molecular phenotypes.

The nonessential amino acid β-alanine was initially identified as a ligand for an orphan GPCR named TGR7 (40), subsequently shown to be Mrgprd (41). β-Alanine specifically activates the Mrgprd receptor, causing extracellular calcium influx and excitation of Mrgprd^+^ neurons (42). There is a functional interaction between Mrgprd and Trpa1, with the latter acting downstream of the former (43). In cultured HEK293 cells, the transfection of Mrgprd alone confers weak responsiveness to β-alanine but not to mustard oil, whereas cotransfection of both Mrgprd and Trpa1 confers strong responsiveness to both ligands (42, 43). Our finding above that eye wipe testing of nociception using mustard oil was unchanged in Rgs7-Cre^+/–^ *Gnb5*^fl/fl^ mice (Figure 4C) suggests Trpa1 function is unaffected by this genetic manipulation. In our experiments with primary cultures of sensory neurons, we therefore used β-alanine as a probe to stimulate Mrgprd function.

**Figure 7:**
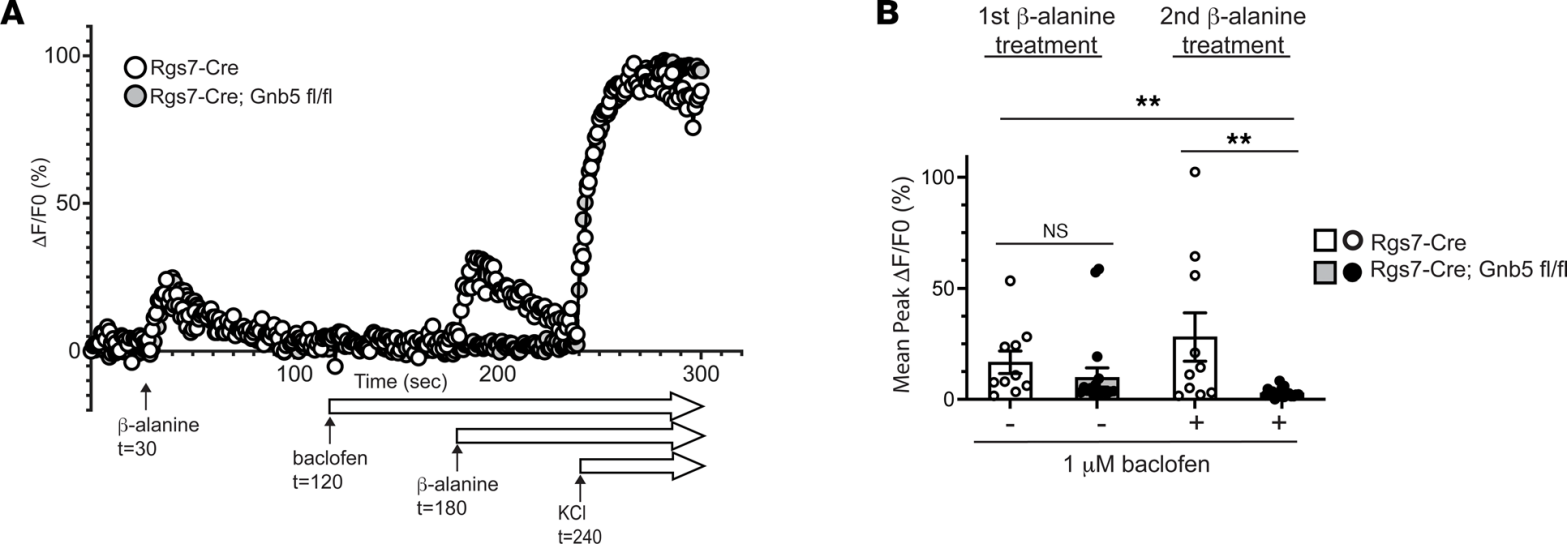
Functional response of primary sensory neurons to activation by the Mrgprd agonist β-alanine before and after treatment with the GABA-B agonist baclofen. Primary cultures of sensory neurons derived from DRG harvested from Rgs7-Cre^+/–^ and Rgs7-Cre^+/–^ *Gnb5*^fl/fl^ mice and transduced in vitro with recombinant adeno-associated virus expressing tdTomato in a Cre-dependent fashion were loaded with the green-fluorescence calcium indicator Fluo-4. Rgs7^+^ cells were identified and studied for responsiveness to treatment with 10 mM of the Mrgprd receptor agonist β-alanine, before and after the addition of 1 μM of the GABA-B receptor agonist baclofen. Experiments were performed in pairs, using primary neurons representing each genotype. (**A**) Tracings of calcium-activated fluorescence from a representative pair of single neurons (1 each from Rgs7-Cre^+/–^ and Rgs7-Cre^+/–^ *Gnb5*^fl/fl^ mice) with the time course and sequence of drug treatment shown. At 240 seconds, 50 mM KCl was added to all cells to induce maximum depolarization. Note that baclofen remained in the bath during the second β-alanine and KCl applications. (**B**) The average peak calcium-activated fluorescence response to the sequential treatments with 10 mM β-alanine, before and after the addition of 1 μM baclofen, derived from 4 experiments are shown. In **B**, the total number of neurons analyzed from Rgs7-Cre^+/–^ mice was 10 (from *n* = 4 mice), and from Rgs7-Cre^+/–^; *Gnb5*^fl/fl^ mice the total number of neurons analyzed was 18 (from *n* = 4 mice). In **B**, the Kruskal-Wallis (KW) test with Dunn’s multiple comparisons testing was employed for all comparisons, with bars indicating mean ± SEM of *n* = 10 neurons (Rgs7-Cre^+/–^ mice) or *n* = 18 neurons (Rgs7-Cre^+/–^ *Gnb5*^fl/fl^ mice). *P* values: KW **P = 0.0014; without baclofen, first β-alanine treatment, Rgs7- Cre^+/–^ control vs. Rgs7-Cre^+/–^ *Gnb5*^fl/fl^, Dunn’s test NS *P* = 0.581; with baclofen, second β-alanine treatment, Rgs7- Cre^+/–^ control vs. Rgs7-Cre^+/–^ *Gnb5*^fl/fl^, Dunn’s test **P = 0.0097.

Three days following rAAV transduction, tdTomato^+^ sensory cells were assayed for intracellular calcium mobilization in response to treatment with the Mrgprd receptor agonist β-alanine, before and after the addition of a low dose of the GABA-B receptor agonist baclofen, by monitoring Fluo-4 fluorescence. Experiments were always performed in pairs, assaying in parallel tdTomato^+^ cells from cultures prepared from Rgs7- Cre^+/–^ and Rgs7-Cre^+/–^ *Gnb5*^fl/fl^ mice. Figure 7A shows the fluorescence response during a typical pair of experiments and the standardized sequence and timing of drug treatment.

Prior to treatment with the GABA-B receptor agonist baclofen, the Mrgprd agonist β-alanine added at the 30-second mark produced similar activation of Rgs7-Cre^+/–^ and Rgs7-Cre^+/–^ *Gnb5*^fl/fl^ cells (Figure 7, A and B). After treatment with 1 μM baclofen, the Rgs7-Cre^+/–^ control neurons responded robustly to the second addition of β-alanine at 180 seconds. In contrast, the same dose of baclofen nearly abolished the response to β-alanine in the Rgs7-Cre^+/–^ *Gnb5*^fl/fl^ neurons, suggesting an enhanced sensitivity to inhibition by the GABA-B agonist (Figure 7, A and B). The pooled responses to the sequential treatments of neurons, taken from 4 pairs of mice, with β-alanine are shown in Figure 7B. These findings are compatible with a model whereby G protein–coupled metabotropic GABA-B receptors, sensitive to regulation by Gβ5 (presumably in complex with Rgs7), modulate the activity of Mrgprd-expressing sensory cells.

## Discussion

Our understanding of how pain signals are processed and regulated inside primary sensory neurons before their projection onto secondary nociceptors at the spinal level is still incomplete. We show here in a series of behavioral, pharmacological, immunohistochemical, and functional experiments that sensitivity to multiple modalities of pain depends on the cellular expression of Gβ5 in primary sensory neurons. Sensitivity to mechanical pain specifically requires Gβ5 in Rgs7-expressing neurons to restrain antinociceptive input from metabotropic GABA-B receptors. The sensory effect of Gβ5 KO from DRG was highly specific since pruriception was unaffected.

The specific implication of Gβ5 and the association of Rgs7 in the regulation of mechanical pain was a striking finding, and our in situ hybridization results suggest an enrichment of Rgs7 in Mrgprd-positive sensory neurons. Since the full expression of mechanical nociception is lost following ablation of Mrgprd-expressing neurons (19), the association of Rgs7 with Mrgprd may make functional sense. The close association of Rgs7 with Mrgprd^+^ sensory neurons further suggests that Rgs7 may be enriched in the same population of nonpeptidergic primary neurons defined also by *P2rx3* (*P2x3*), *Gfra2*, and *Ret* expression (20). A study of Mrgprd^+^ sensory neurons by Reynders et al. revealed high levels of Rgs7 expression in the IB4^+^ population of unmyelinated C-fibers (44). Future deep transcriptional profiling of Rgs7^+^ sensory neurons should enable the confirmation and clarification of the receptors and other molecular markers that define this population in greater detail.

Metabotropic GABA-B receptors play an important role in the modulation of pain. However, relatively little is known about the function of these receptors in primary sensory neurons that mediate the primary nociceptive signal (29, 30). There is an abundance of GABAergic inhibitory interneurons in laminae I–III of the mouse spinal dorsal horn (45), suggesting that sensory afferents expressing metabotropic GABA-B receptors in their nerve terminals could be sensitive to GABAergic inhibition. Experiments in mice have shown that GABA-B receptor activation causes presynaptic inhibition of a population of nonpeptidergic nociceptors (46). Our results, which show that targeted knockout of Gβ5 from sensory cells potentiated the analgesic effects of GABA-B agonists on mechanical pain, would be consistent with a mechanism of GABA-B receptor–mediated presynaptic inhibition.

The present work that links Rgs7, Gβ5, and GABA-B receptor function within primary mechanical nociceptors is not the first to associate the regulation of metabotropic GABA-B receptors with Gβ5 and Rgs7. Xie and coworkers reported that Gβ5, in complex with Rgs7 and/or another R7-RGS protein, influenced the timing and sensitivity of the GABA-B receptor/GIRK signaling pathway in mouse hippocampal neurons (39). The physical association of Gβ5/Rgs7 with GABA-B receptors was subsequently documented in hippocampal CA1 pyramidal neurons (33). Ostrovskaya and coworkers have also shown that in the hippocampus, Gβ5/Rgs7 regulates synaptic plasticity and memory via the modulation of inhibitory GABA-B receptor signaling (34, 35). Targeted knockout of Rgs7 from sensory neurons will be required to confirm the hypothesis suggested by our data that Rgs7, as part of a Gβ5/Rgs7 complex downstream of GABA-B receptors, might specifically regulate mechanical nociception.

Only hot plate analgesia was affected by targeted KO of *Gnb5* from Rgs9^+^ cells. Global KO of Rgs9 in mice has previously been shown to affect hot plate analgesia and to potentiate the analgesic effects of mu opioid receptor agonists (23). It remains unclear from our experiments, therefore, whether the hot plate analgesia resulting from targeted KO of Gβ5 from sensory neurons involves destabilization and loss of Rgs9 normally expressed in these afferents or, conversely, the hot plate analgesia resulting from targeted KO of *Gnb5* from Rgs9^+^ cells is a peripheral or central effect, or some combination thereof. With respect to the possible central involvement of Rgs9, the expression of Rgs9 has been demonstrated in central pain-regulatory regions, including the periaqueductal gray, striatum, and the dorsal horn of the spinal cord (22, 23, 47). Such expression depends on Gβ5, since Rgs9 expression in the central nervous system is no longer demonstrable following KO of Gβ5, as a consequence of Rgs9’s destabilization (7).

The observation that the targeted KO of *Gnb5* from Rgs9^+^ cells affected thermal but not capsaicin-mediated nociception raises the intriguing possibility that non-Trpv1 pathways in the periphery may be involved. Transient receptor potential melastatin-3 (Trpm3) is a thermosensor expressed in both Trpv1-expressing and Trpv1–non-expressing sensory neurons (48). Trpm3^−/−^ mice exhibit a strong deficit in the detection of noxious heat stimuli, consistent with a role for Trpm3 as a nociceptor channel involved in acute heat sensing (48). In light of our present findings, it is interesting that GABA-B receptor activation has been previously shown to inhibit TRPM3 (49, 50). Whether or not normal Trpm3 function in Rgs9^+^ neurons may depend on Gβ5 expression remains to be explored.

We were not able to recapitulate the loss of nociception observed in Advillin-Cre^+/–^ *Gnb5*^fl/fl^ mice during eye wipe testing after treatment with capsaicin by the cKO of Gβ5 from either Rgs7^+^ or Rgs9^+^ neurons. Trpv1 is the molecular sensor for capsaicin, and eye wipe testing depends on corneal sensitivity. The cornea is innervated by the ophthalmic branch of the trigeminal nerve, whose cell bodies reside in the trigeminal ganglion (TRG), not the spinal sensory ganglia. Though we did not analyze the coexpression of Trpv1 and Rgs9 in the TRG in this study, prior single-cell transcriptomic profiling of mouse TRG cells showed that approximately 27% of Rgs7^+^ and 45% of Rgs9^+^ TRG neurons coexpress Trpv1 (51). Thus, the failure to show any loss of nociception to the ocular application of capsaicin following cKO of Gβ5 from Rgs7^+^ or Rgs9^+^ neurons was perhaps unexpected. This leaves open the possibility that this regulatory action of Gβ5 involves Rgs6^+^ and/or Rgs11^+^ neurons that do not also coexpress Rgs7 or Rgs9. The loss-of-nociception phenotype would still be evident following Gβ5 KO because all 4 R7-RGS proteins would be lost through destabilization.

Neuropathic pain can result from diseases of the peripheral nervous system that disrupt somatosensation. Painful diabetic polyneuropathy is a striking example that affects millions worldwide. Too many patients who suffer from chronic pain, including those with painful neuropathies, are at risk for opioid dependence given the lack of effective alternative analgesics. This underscores an urgent public health need to identify alternative drugs that can regulate nociceptive pathways and target the intracellular downstream targets shared with opioid receptors.

Our findings link Rgs7, Gβ5, and inhibitory GABA-B receptor function within primary mechanical nociceptors. These results suggest that the targeted inhibition of Gβ5 function in Rgs7-expressing neurons by drugs or small molecule inhibitors might provide specific relief for mechanical allodynia via the amplification of the endogenous antinociceptive action of metabotropic GABA-B receptors. Furthermore, our findings suggest that such targeted inhibitors of Gβ5 function might effectively synergize with low therapeutic doses of GABA-B agonists, possibly circumventing some of the sedative side effects of the latter agents that have limited their systemic use for analgesia. The discovery that Gβ5 regulates intracellular pain signaling in sensory neurons further establishes the foundation for the possible localized application of future analgesics targeting Gβ5 and GABA-B receptor–regulated afferent signaling pathways, with the potential of minimizing side effects associated with systemic drug administration.

## Methods

### Mouse husbandry and genotyping.

Mice were housed and treated in strict accordance with the NIH *Guide for Care and Use of Laboratory Animals* (National Academies Press, 2011). Age-matched male and female mice from littermate cohorts, 3 to 10 months of age, were used for all the experiments.

To conditionally inactivate the *Gnb5* gene located on chromosome 9, 2 tandem *loxP* sites were engineered to flank exon 3 in C57BL/6 mice (7, 52) and used in this study. The forward primer 5′-TTACATGTTCCAAGTCACAGCC-3′ and the reverse primer 5′-AAGAGCCTGCTGGTGAAGGTGC-3′ were used for PCR genotyping with product sizes of ~210 bp for wild-type allele and ~250 bp for floxed allele, respectively.

Advillin-Cre mice were a gift from Fan Wang, Duke Neurobiology Program, Duke University School of Medicine, Durham, North Carolina, USA (14, 15). Mice harboring an Ai9-Cre reporter allele were from The Jackson Laboratory (stock no: 007909) (17). Rgs9-Cre knockin mice were a gift from Yuqing Li, Department of Neurology, College of Medicine, University of Florida, Gainesville, Florida, USA (21). Rgs7-Cre knockin mice were generated by insertion of an internal ribosome entry site and Cre recombinase coding sequence following the stop codon present in exon 17 of Rgs7 (reference EMBL-EBI OTTMUST00000127638; Ensembl ENSMUST00000192227.5) on chromosome 1, followed by excision of the neomycin selection cassette via flippase recombinase (Ozgene Pty Ltd.).

### Behavioral testing.

For all behavioral testing the genotype of the mouse being tested was masked. Age- and sex-matched mice from littermate cohorts, 3 to 10 months of age, were used for all the experiments. All behavioral testing was performed during the light cycle (daylight hours).

For the experiments shown in Figure 1, for panels D–K, *n* = number of mice from each genotype tested (for panel D, *Gnb5*^fl/fl^ *n* = 6, Advillin-Cre^+/–^ *Gnb5*^fl/fl^ *n* = 6; for panel E, *Gnb5*^fl/fl^ *n* = 8, Advillin-Cre^+/–^ *Gnb5*^fl/fl^ *n* = 7; for panel F, *Gnb5*^fl/fl^ *n* = 7, Advillin-Cre^+/–^ *Gnb5*^fl/fl^ *n* = 5; for panel G, *Gnb5*^fl/fl^ *n* = 6, Advillin-Cre^+/–^ *Gnb5*^fl/fl^ *n* = 6; for panel H, *Gnb5*^fl/fl^ *n* = 10, Advillin-Cre^+/–^ *Gnb5*^fl/fl^ *n* = 9; for panel I, *Gnb5*^fl/fl^ *n* = 12, Advillin-Cre^+/–^ *Gnb5*^fl/fl^ *n* = 9; for panel J, *Gnb5*^fl/fl^ *n* = 8, Advillin-Cre^+/–^ *Gnb5*^fl/fl^ *n* = 8; for panel K, *Gnb5*^fl/fl^ *n* = 11, Advillin-Cre^+/–^ *Gnb5*^fl/fl^ *n* = 11). For the experiments shown in Figure 5, for panels A–D, *n* = number of mice from each genotype tested (for panel A both control and 2-hydroxysaclofen *Gnb5*^fl/fl^ *n* = 7, Advillin-Cre^+/–^ *Gnb5*^fl/fl^ *n* = 9; for panel B control, *Gnb5*^fl/fl^ *n* = 12, Advillin-Cre^+/–^ *Gnb5*^fl/fl^ *n* = 9, for panel B 2-hydroxysaclofen, *Gnb5*^fl/fl^ *n* = 8, Advillin-Cre^+/–^ *Gnb5*^fl/fl^ *n* = 9; for panel C control, *Gnb5*^fl/fl^ *n* = 10, Advillin-Cre^+/–^ *Gnb5*^fl/fl^ *n* = 11, for panel C 2-hydroxysaclofen, *Gnb5*^fl/fl^ *n* = 8, Advillin-Cre^+/–^ *Gnb5*^fl/fl^ *n* = 9; for panel D control *Gnb5*^fl/fl^ *n* = 11, Rgs7-Cre^+/–^ *Gnb5*^fl/fl^ *n* = 10, for panel D 2-hydroxysaclofen *Gnb5*^fl/fl^ *n* = 10, Rgs7-Cre^+/–^ *Gnb5*^fl/fl^ *n* = 9). For the experiments shown in Figure 6, for panels A–D, *n* = number of mice from each genotype tested: for panel A pre-CFA, *Gnb5*^fl/fl^ *n* = 11, Advillin-Cre^+/–^ *Gnb5*^fl/fl^ *n* = 11; for panel A post-CFA and panel C, all doses, *Gnb5*^fl/fl^ *n* = 12, Advillin-Cre^+/–^ *Gnb5*^fl/fl^ *n* = 12; the 12 mice used for panel A post-CFA and panel C represent the identical 11 mice used for panel A pre-CFA plus 1 additional mouse; for panel B pre-CFA, *Gnb5*^fl/fl^ *n* = 8, Rgs7-Cre^+/–^ *Gnb5*^fl/fl^ *n* = 8; for panel B post-CFA and panel D, all doses, *Gnb5*^fl/fl^ *n* = 10, Rgs7-Cre^+/–^ *Gnb5*^fl/fl^ *n* = 11; the panel B post-CFA and panel D mice represent the identical 8 mice used for panel B pre-CFA plus 2 (*Gnb5*^fl/fl^) or 3 (Rgs7-Cre^+/–^ *Gnb5*^fl/fl^) additional mice.

### Testing for nociception.

Hot plate analgesia using a 53°C hot plate apparatus (Columbus Instruments) was evaluated as previously described (53). Animals were habituated in the room for 1 hour in the testing room prior to testing for latency to jump or hind paw lick after placing it on the heated plate. Each animal was tested 3 times at an interval of 5 minutes. A cutoff time of 40 seconds was used to avoid tissue damage or inflammation.

Von Frey testing for static mechanical algesia was performed as previously described (54). The estimation of mechanical withdrawal threshold for assessing mechanical algesia was performed using the ascending stimulus method using a set of calibrated von Frey filaments. Mice were placed in the testing room at least 1 hour prior to testing on an elevated wire mesh. Each von Frey filament was applied manually in perpendicular fashion to the plantar surface of either hind paw until the filament buckled, delivering a constant predetermined force. A response was considered positive if the mouse exhibited any nocifensive behaviors, including brisk paw withdrawal, licking, or flinching, either during application of the stimulus or immediately after the filament was removed. Von Frey monofilaments were applied with increasing force until a withdrawal response was elicited, and the force of the von Frey filament that elicited this positive response was designated as the mechanical withdrawal threshold. A positive response to a filament was noted when the animal responded 3 times by withdrawal in 5 applications. The total elapsed testing time during monofilament application varied due to activity level and sensitivity of the mice. Manual von Frey testing was performed by a person skilled in this test who could differentiate the nocifensive behavior of mice from normal grooming or movement of hind limbs.

Eye wipe assays were performed as described by Mishra et al. (55). Chemical sensitivity of the ophthalmic branch of the trigeminal nerve was investigated using drops (50 μL) of diluted capsaicin (100 μM, MilliporeSigma No. M2028) or mustard oil–allyl isothiocyanate (10 μM, MilliporeSigma No. W203408) dissolved in PBS to either eye and counting the number of wipes for 1 minute.

Nociceptive testing following pharmacologic blockade of the GABA-B receptors was performed using intraperitoneal (i.p.) administration of 5 mg/kg 2-hydroxysaclofen (Tocris, catalog 0245) 30 minutes before testing. Saline was administered i.p. as control.

### Behavioral responses to intradermal and intrathecal pruritogens.

The scratch response to intradermal injection of pruritogens was assayed as previously described by Sun et al. (56) and as modified (13). The pruritogens employed were of the highest purity commercially available and included the classical pruritogens compound 48/80 (100 μg/10 μL) (MilliporeSigma, catalog C2313) and chloroquine diphosphate salt (200 μg/10 μL) (MilliporeSigma, catalog C6628).

The scratch response to the intrathecal drug injection of pruritogens was performed as described by Hylden and Wilcox (57) with modifications as recently described by Pandey et al. (13). Penetration of the lumbar spinal dura mater by the needle was confirmed by the classic tail flick response (58). Scratching responses were monitored over a period of 45 minutes following injection. Peptides for intrathecal testing were of the highest purity commercially available and included GRP (1–5 nmol) (Bachem, catalog H3120) and Nppb (1–5 nmol) (MilliporeSigma, catalog B9901) (59). Individual mice were used for the testing of no more than 2 intrathecal pruritogens in the behavioral study, with at least 15 days of rest/washout in their home cage between tests.

### Subacute inflammatory pain model.

Induction of inflammatory pain was performed as described (60), following the intraplantar injection of CFA (MilliporeSigma, catalog F5881) (20 μL) into the mouse hind paw. Mechanical hyperalgesia was then assessed 24 hours following the paw injection, after the i.p. administration of either saline or different doses of baclofen (Tocris, catalog 0796), a GABA-B receptor agonist. The baclofen was dissolved in saline (0.5, 2, and 5 mg/kg for Advillin-Cre *Gnb5*^fl/fl^ mice and 0.5, 1, and 2.5 mg/kg for Rgs7-Cre *Gnb5*^fl/fl^ mice). Mice were acclimatized for 30 minutes after the i.p. administration of saline or a given dose of baclofen, following which the CFA-injected hind paw was tested with von Frey filaments.

### Functional studies of sensory neurons in primary culture using Fluo-4.

Age- and sex-matched mice with Rgs7-Cre^+/–^ *Gnb5*^fl/fl^ and control Rgs7-Cre^+/–^ genotypes were anesthetized with avertin and decapitated. The DRG were rapidly removed and collected and rinsed twice in cold HBSS. DRG were then sequentially digested by treatment with papain, neutral protease (dispase) (Worthington Biochemical Corporation), and collagenase type II (Gibco) for 10 minutes each at 37°C. After digestion, isolated DRG were resuspended in 400 μL of culture media (Neurobasal medium, supplemented with B-27 and GlutaMAX; Thermo Fisher Scientific) and plated onto poly-d-lysine– and laminin-coated chambered coverslips (Ibidi). The cultured DRG were transduced with an rAAV construct designed to express tdTomato in a Cre-dependent fashion, due to the location of a *loxP*-flanked transcriptional termination element (“stop” sequence) between the promoter and tdTomato coding sequence (Addgene, catalog 100048-AAV1). Following rAAV transduction, the cells were grown on coverslips for 3 days and then assayed for intracellular calcium mobilization, as described below, using a Fluo-4 Assay Kit (Abcam, ab228555).

Prior to the assay, cell culture media were removed and replaced with Fluo-4 dye dissolved in HBSS media. The cells were then incubated at 37°C for 60 minutes, following the manufacturer’s instructions. After incubation, tdTomato^+^ cells were assayed for intracellular calcium mobilization, employing a Leica Sp8 confocal microscope equipped with a 20× NA 1.4, oil immersion objective for live confocal microscopy and video capture. During live video capture of the calcium flux enhanced fluorescence, the cells were treated according to the following 300-second protocol: *t* = 0 seconds, begin baseline recording; *t* = 30 seconds, treatment with 10 mM β-alanine (Mrgprd agonist); *t* = 120 seconds, treatment with 1 μM baclofen (GABA-B receptor agonist); *t* = 180 seconds, second treatment with 10 mM β-alanine; and *t* = 240 seconds, neurons were depolarized by treatment with 50 mM KCl.

Images were captured and analyzed for fluorescence intensity changes, as a reflection of changes in the bound versus unbound calcium state, as previously described (61). Quantification of changes in fluorescence intensity over the 5 minutes of the assay employed Imaris software (version 9.3.1) (Oxford Instruments) and was expressed as percentage change in fluorescence intensities (F/F_0_) and plotted using Prism 8, Version 8.1.2 (GraphPad Software, Inc.). The experiment was performed and quantitation completed while the genotype of the mice from which DRG originated was masked.

### IHC and histofluorescence.

Mice were anesthetized with avertin and perfused with 10 mL of PBS followed by 4% paraformaldehyde. The mice were immediately transferred to Vitrovivo Biotech for further processing. Briefly the spinal column was bisected from one end to the other with scissors. DRG (*n* = 8–12) were dissected from each mouse spinal column and incubated in 30% sucrose for 24 hours before they were embedded in OCT (Scigen, Inc.). Frozen sections were cut at a thickness of 10 μm and fixed with cold acetone/methanol mixture (1:1) for 15 minutes. The sections were first blocked with normal goat serum in PBS and then incubated with primary antibodies at 4°C overnight. After washing, the slides were incubated with secondary antibody at room temperature for 60 minutes and counterstained with DAPI for 2 minutes. Sections were mounted with VECTASHIELD Antifade Mounting Medium (Vector Labs), coverslipped, and imaged. Isotype controls for different primary antibodies used were normal guinea pig (Sino Biological Inc.), rabbit polyclonal IgG (BioLegend), and normal chicken IgG (Leinco Technologies, Inc.). The sources and dilutions of the antibodies employed for the IHC and immunohistofluorescence studies are listed in Table 1. The percentage of residual antibody signal following Advillin-Cre–mediated knockout of *Gnb5* was calculated in 4 steps: 1. Counting of immunopositive cells in DRG harvested from *Gnb5*^fl/fl^ control and Advillin-Cre^+/–^ *Gnb5*^fl/fl^ mice and stained with DAPI (*n* = 3 DRG sections for each genotype; each section counted 3 separate times); 2. Determination of the areal density of immunopositive cells by division of the raw cell count by the area of the DRG, as defined by DAPI staining; 3. Normalization of the data by division of all values by the mean *Gnb5*^fl/fl^ control value; 4. Calculation of the mean ± SEM residual antibody signal in Advillin-Cre^+/–^ *Gnb5*^fl/fl^ mice as a percentage (results shown below the middle panels of the images in Figure 1, A–C).

### RNAscope analysis.

RNAscope analysis of gene transcripts in mouse DRG was performed using the RNAscope HiPlex v2 assay kit (ACDbio) according to the manufacturer’s instructions for fresh frozen sections by employing the 30-minute proteinase III treatment option. This platform allowed serial detection of 12 genes from the same DRG section in 4 rounds with 3 genes detectable each round. The gene probes and the corresponding image colors used in this study are listed in Table 2. Images for each gene probe were taken using a KEYENCE All-in-One Fluorescence Microscope, BZ-X800/BZ-Z810. The images were first aligned based on DAPI-stained nuclei by the Image Registration software (ACDbio) and analyzed using the CellProfiler software (62). Only signals detectable in nuclei were counted and quantified for each cell after reference to the background sections simultaneously processed with nonspecific probes as negative controls. A total of 5,200 cells from 5–7 DRG per each of 3 mice were analyzed. The percentage of 5,200 analyzed cells expressing and/or coexpressing different genes was calculated using Microsoft Excel. The clustering of gene expression patterns in different DRG were analyzed by the UMAP algorithm (26) employing Has2k1/Plotnine software v0.8.0 that ran on the Jupyter Notebook interface of Python 3 (63).

### Statistics.

No sample size calculation was used to predetermine sample sizes. Sample size was chosen as a balance between establishing confidence in reproducibility (on one hand) and practical considerations, such as the time required to breed animals of a particular age, sex, and genotype (on the other). Sample size is reported in the legend to each figure. Data normality was tested by both the Shapiro-Wilk and Kolmogorov-Smirnov methods. In most cases, the results of the 2 methods were congruent. If a data set failed 1 or both tests of normality, that data set was assumed to follow a non-Gaussian distribution, and nonparametric statistical tests were employed. For comparison of normal data sets, the 2-tailed unpaired Student’s *t* test or 1-way ANOVA with Tukey’s multiple comparisons testing was employed, as appropriate. For comparison of data sets that included non-Gaussian data, the Mann-Whitney or Kruskal-Wallis with Dunn’s multiple comparisons testing was employed, as appropriate. No data points were excluded. Significance level was set at *P* < 0.05 and all data are reported as mean ± SEM. Prism 9 computer software, Version 9.5.1 (GraphPad Software, Inc.), was employed for the statistical analysis.

### Study approval.

All mouse experiments were conducted under the auspices of the NIH National Institute of Diabetes and Digestive and Kidney Diseases Animal Care and Use Committee, protocol K164-MDB-20, in accordance with the US Department of Agriculture Animal Welfare Act.

## Author contributions

MP, JHZ, PRA, and WFS designed and analyzed results of the behavioral, IHC, and histofluorescence studies. MP, JHZ, PRA, CK, NL, AA, NJ, RT, KD, JS, and ABK performed the behavioral, IHC, histofluorescence, and in situ hybridization studies. MP, JHZ, SG, and JK designed and performed the mouse DRG Ca^2+^ imaging studies. CKC designed and generated the *Gnb5*-floxed mouse model and provided critical intellectual input. All authors contributed to the discussion and interpretation of the results. WFS wrote the manuscript, with contributions and suggestions from all authors.

## Supplementary Material

Supplemental data

## Figures and Tables

**Table 1:**
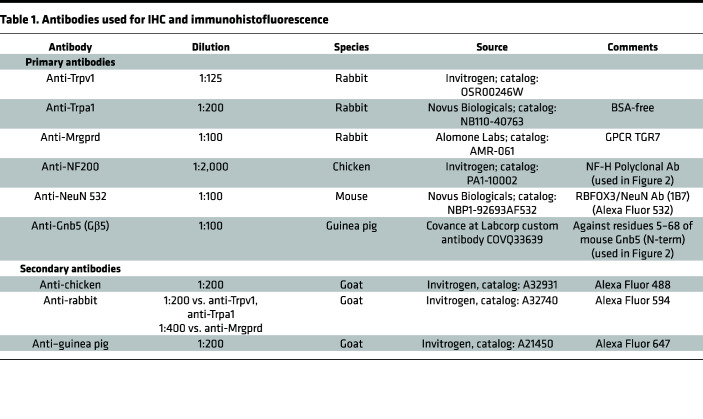
Antibodies used for IHC and immunohistofluorescence

**Table 2:**
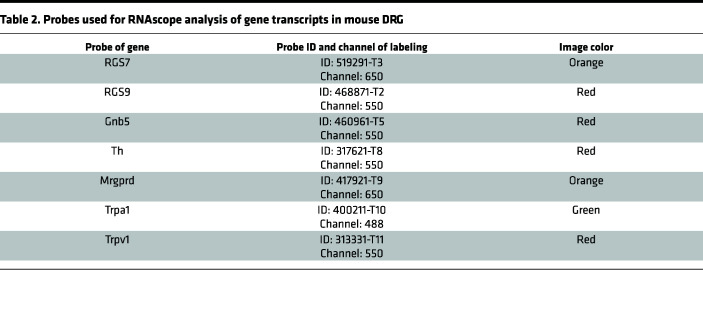
Probes used for RNAscope analysis of gene transcripts in mouse DRG

## Version 1 05/23/2023

In-Press Preview

## Version 2 07/10/2023

Electronic publication
