# Supplementary material for: Specific regulation of mechanical nociception by Gβ5 involves GABA-B receptors
Source: JCI Insight. 2023 Jul 10;8(13):e134685. doi: 10.1172/jci.insight.134685 (PMC10371342; doi:10.1172/jci.insight.134685)
Supplement: Supplemental data [file jciinsight-8-134685-s028.pdf]

Supplemental Data for
"**Specific regulation of mechanical nociception by Gβ5 involves GABA-B receptors**", by Pandey *et al*

Table of Contents

Supplemental Fig. 1. Presence of Rgs7-Cre and Rgs9-Cre alleles in the germline does not impair nociception.

Supplemental Fig. 2. Validation of Rgs9-Cre and Rgs7-Cre mouse lines by crossbreeding with Ai9 Cre reporter strain and antibody staining of dorsal root ganglia.

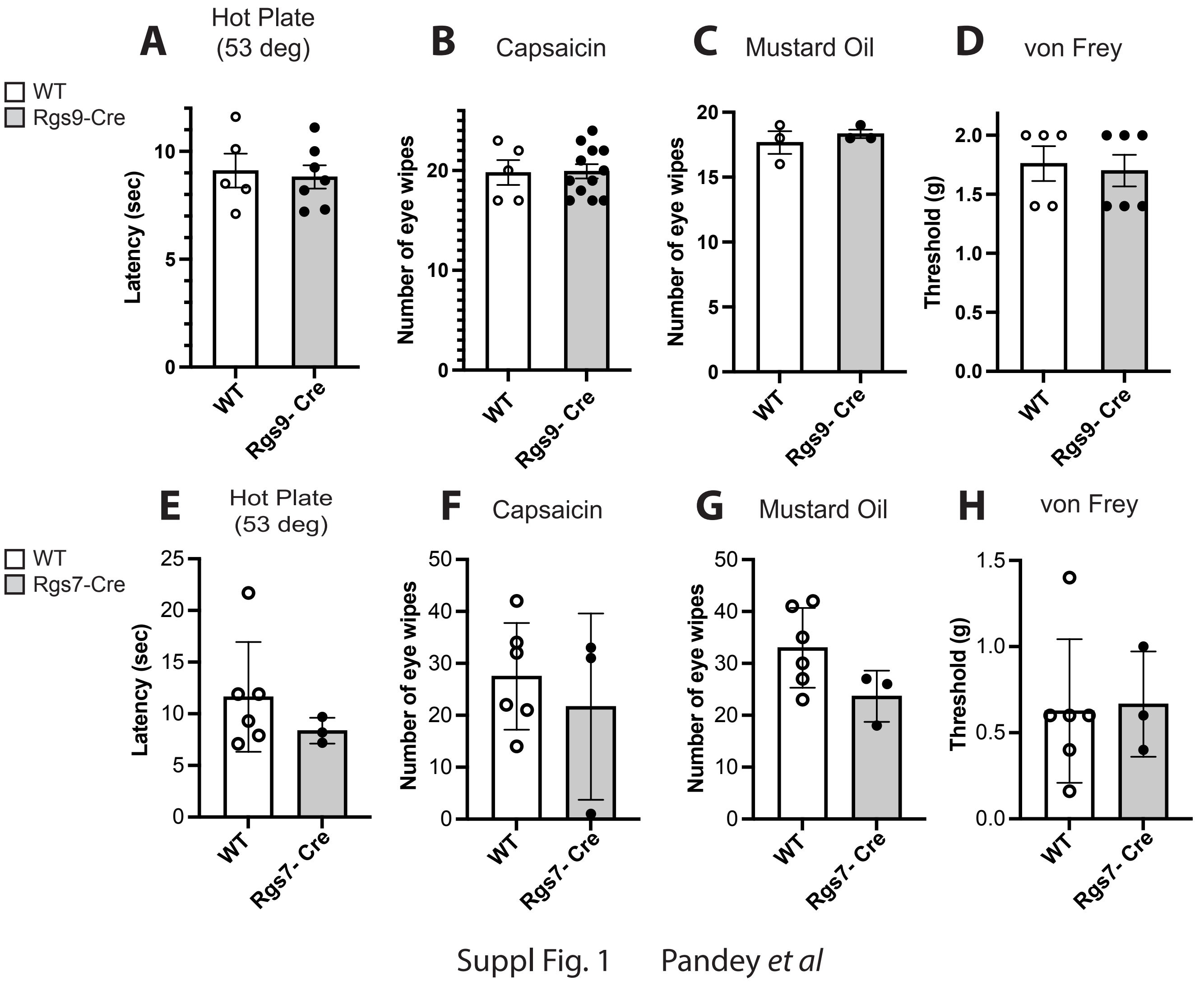

Supp. Fig. 1. Presence of Rgs7-Cre and Rgs9-Cre alleles in the germline does not impair nociception. Behavioral testing of nociception in littermates with either the wild-type **(A-H),** Rgs9-Cre+/- **(A-D)**, or Rgs7-Cre+/- **(E-H)** genotypes, as indicated. **(A, E)** Hot plate testing (53o C). **(B, F)** Eye wipe testing using the Trpv1 agonist capsaicin. **(C, G)** Eye wipe testing of chemical nociception using the Trpa1 agonist mustard oil. **(D, H)** Von Frey filament behavioral testing of mechanical nociception. For A-H, the evaluator was blind to the genotype of the mouse being tested. In A and B, the two-tailed unpaired Student's t-test was employed, and for C-H the Mann-Whitney test was utilized, with bars indicating mean ± S.E.M. For panels A-H, n= number of mice from each genotype tested (for panel A, WT n= 5, Rgs9-Cre+/- n=7; for panel B, WT n= 5, Rgs9-Cre+/- n=12; for panel C, WT n= 3, Rgs9-Cre+/- n=3; for panel D, WT n= 5, Rgs9-Cre+/- n=6; for panel E, WT n= 6, Rgs7-Cre+/- n=3; for panel F, WT n= 6, Rgs7-Cre+/- n=3; for panel G, WT n= 6, Rgs7-Cre+/- n=3; for panel H, WT n= 6, Rgs7-Cre+/- n=3. P values: A-H, all > 0.05.

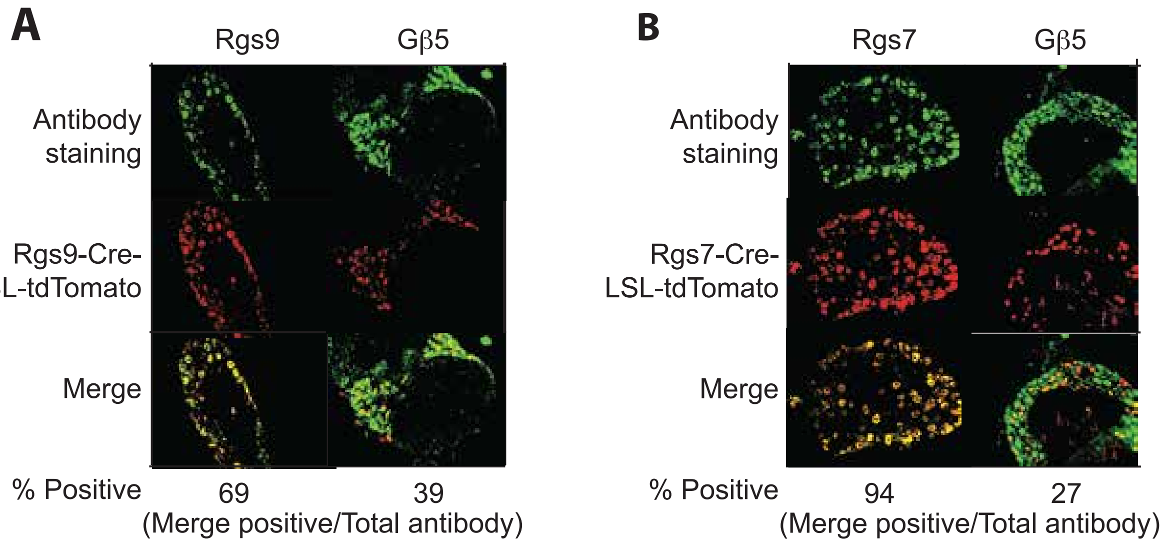

Supplemental Fig. 2 Pandey *et al*

Supp. Fig. 2. Validation of Rgs9-Cre and Rgs7-Cre mouse lines by crossbreeding with Ai9 Cre reporter strain and antibody staining of dorsal root ganglia. (A) Sections through lumbar dorsal root ganglia (DRG) harvested from Rgs9-Cre+/-; Ai9 (LSL-tdTomato) mice and analyzed by dual immunohistochemistry (IHC) and histofluorescence. Red fluorescence indicates tdTomato expression present in a subset of sensory neurons. Analysis by IHC of the same DRG sections with antibodies to Rgs9 and Gbeta5 (green signal). **(B)** Sections from DRG harvested from Rgs7-Cre+/-; Ai9 mice, with red fluorescence indicating tdTomato expression in a subset of sensory neurons. Analysis by IHC of the same DRG sections with antibodies to Rgs7 and Gbeta5 (green signal). The percentage antibody-positive DRG neurons co-expressing the Cre-dependent tdTomato signal relative to total antibody-positive cells is indicated below each set.
